# Supplementary material for: The Human Genetic Differences in the Outcomes of mRNA Vaccination against COVID-19: A Prospective Cohort Study
Source: Vaccines (Basel). 2024 Jun 5;12(6):626. doi: 10.3390/vaccines12060626 (PMC11209249; doi:10.3390/vaccines12060626)
Supplement: Supplementary file 1 [file vaccines-12-00626-s001.zip › vaccines-3002035-supplementary.pdf]

Supplementary materials

Table S1. Power calculation.

Model	Test	True	MAF	OR	Power	Case.Rate	n per group
Model 1	Additive	Additive	0.407	2.991	0.8	0.5	32
Model 2	Additive	Additive	0.407	3.579	0.8	0.5	25

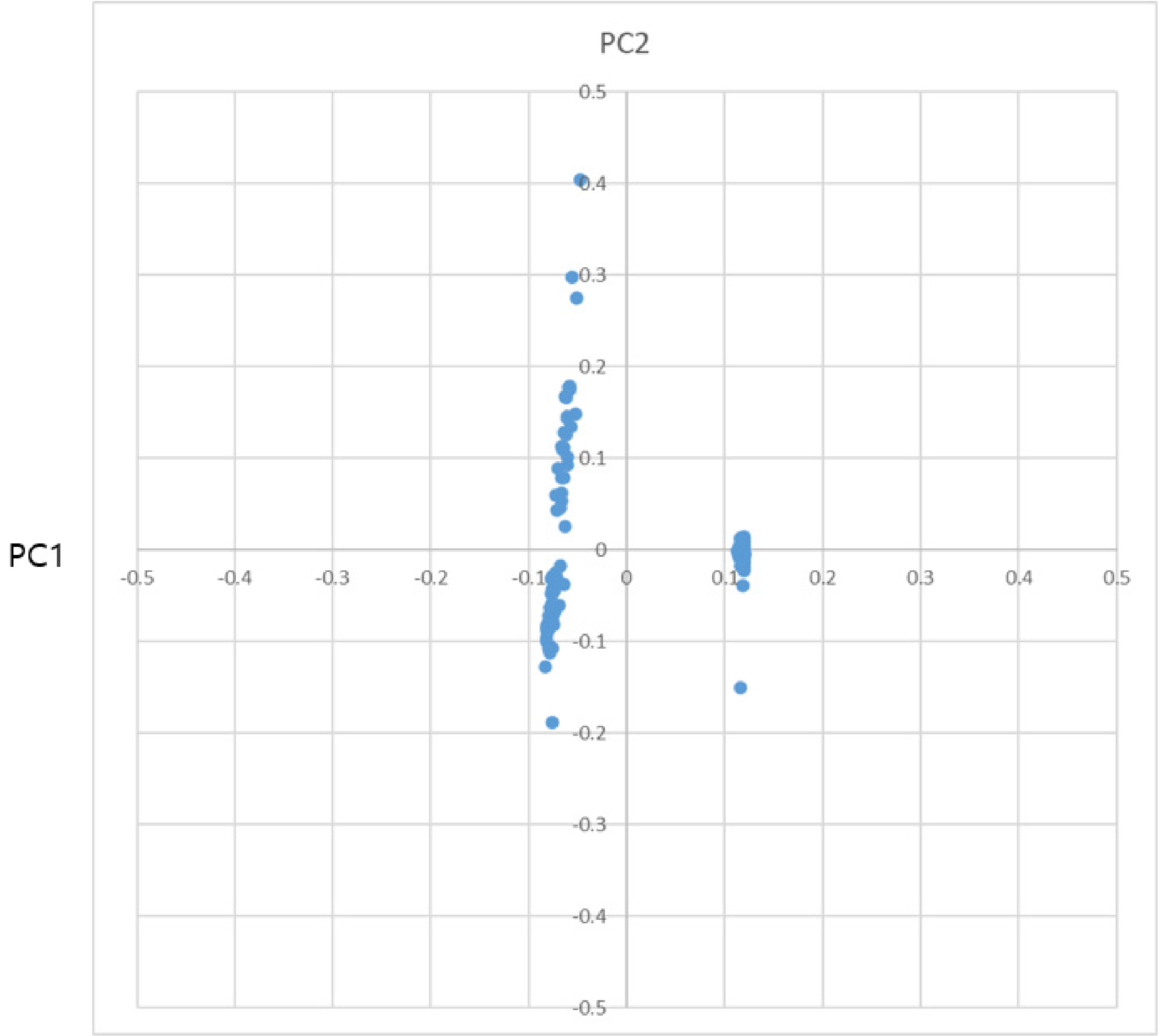

Figure S1. PC1 the first principal component, PC2 the second principal component.
